# Recreational Use of Spa Thermal Waters: Criticisms and Perspectives for Innovative Treatments

**DOI:** 10.3390/ijerph15122675

**Published:** 2018-11-28

**Authors:** Federica Valeriani, Lory Marika Margarucci, Vincenzo Romano Spica

**Affiliations:** Public Health Unit, University of Rome “Foro Italico”, Rome 00135, Italy; federica.valeriani@uniroma4.it (F.V.); lory.margarucci@tiscali.it (L.M.M.)

**Keywords:** recreational water, spa, thermal water, innovative treatment

## Abstract

Natural spa springs are diffused all over the world and their use in pools is known since ancient times. This review underlines the cultural and social spa context focusing on hygiene issues, public health guidelines and emerging concerns regarding water management in wellness or recreational settings. The question of the "untouchability" of therapeutic natural waters and their incompatibility with traditional disinfection processes is addressed considering the demand for effective treatments that would respect the natural properties. Available strategies and innovative treatments are reviewed, highlighting potentials and limits for a sustainable management. Alternative approaches comprise nanotechnologies, photocatalysis systems, advanced filtration. State of the art and promising perspectives are reported considering the chemical-physical component and the biological natural complexity of the spa water microbiota.

## 1. Introduction

Natural spa springs are used for recreational purposes or wellness applications and are available globally [1,2,3]. Especially in the Mediterranean basin, these waters have been exploited and valorized for health and recreational purposes since ancient times [4,5,6,7]. After inheriting the approach to health and well-being from the Greek culture, the Romans magnified this opportunity of personal and social care through the realization of the monumental *thermae publicae*, with major spa buildings that included areas for baths, gardens, stadiums, gyms, restrooms and spaces for massages or health-related activities [7]. Over the centuries and different cultures, spas have maintained a significant role for promoting health in the community. Nowadays, the increase in wellness awareness and fitness expectations has led to the exploitation of thermal waters and extended spa businesses, based on the notion of a joint interaction between natural resources and manmade enterprises [8]. Collectively, the spa economy is estimated at $94 billion, with a consistent growth perspective in the coming decades [9]. Indeed, the global wellness economy had amplified the demand and the offer of products or services based on mineral waters, sea and hot spring resources [8,9]. Specifically, the spa and recreational thermal water tourism mainly flows towards Europe, mostly in German-speaking and Mediterranean countries, but also in North America and Southeast Asia [8,9,10,11]. The application of thermal waters in swimming pools, spa and wellness centers represents a renewed and promising tool for prevention, rehabilitation, and health promotion, providing possible physical, mental or social benefits to patients and several groups of people [1,12]. The general context of spa environments can support a holistic approach to health promotion, also through the exposure to natural open-spaces, the presence of water itself, the access to physical activities, physiotherapies, and health education opportunities. Even if additional evidence-based data are needed, several studies have shown the therapeutic role of mineral elements and other chemical compounds present in thermal waters [13]. The treatments with mineral thermal water or mud proved effective in pain relief and function restoration, impacting also on quality of life: several parameters of clinical interest, and other key issues were reported to play a role in several rheumatologic diseases e.g., knee and hand osteoarthritis, chronic low back pain, rheumatoid arthritis, and osteoporosis) compared to baseline and non-mineral similar treatments [14,15,16]. The thermal waters and spas have ancient roots in history and still today represent a promising opportunity for physical and social well-being but require surveillance to assure appropriate hygiene standards to the final aim of reducing hazards and maximizing benefits.

The water of natural spas should be of satisfactory microbiological quality and must be adequately managed to control the exposure of bathers and personnel to infectious agents. Indeed, the literature describes individual cases or outbreaks associated with the use of swimming/spa pools or similar environments, such as hot springs, hot tubs, whirlpools, natural spas, for recreational, wellness or therapeutic purposes [17,18,19,20,21]. Knowledge on pool uses and on composition of the water that supplies the spa is needed for an effective and appropriate management. Indeed, the peculiar and typical composition of each thermal water represents an interesting richness and a potentially beneficial property for health, but it also implies additional difficulties in defying the correct management, treatment and monitoring of that specific water in a defined application, such as aerosol, beverages lavender or in pool [22,23]. Based on their geological composition, natural waters may be enriched with several salts and ions, such as sulfur, halogens from group 17 of the periodic table, e.g., chlorine (Cl), bromine (Br), iodine (I), or alkaline earth metals comprising group 2 of the periodic table, e.g., magnesium (Mg) or calcium (Ca) [23,24]. Therefore, in natural spa pools, the water should be left untreated for assuring the specific composition, maintaining the original properties and the potential health benefits [10,11,24,25,26,27]. However, it is well known that pools and spas can present a considerable source of infection and other threats to human health [22,28,29]. In particular, several bacteria such as *Legionella, Pseudomonas, Mycobacteria*, as well as protozoa such as amoebae, algae and other microorganisms can naturally proliferate in the conditions characteristic of thermal waters and, if not managed properly, can become a hazard for users [17,18,19,20,21]. This problem represents a dilemma between treating or not treating natural spa waters and induces several pool managers to add disinfectants into the natural solutions highly rich in salts, resigning the original water properties in favor of safety, even if there is a lack of knowledge on the chemical risks related to the use of disinfectants in these waters. Several alternative strategies have been proposed and the recent progress in nanotechnologies is contributing to the field, leading to the introduction of innovative water treatment strategies for thermal waters and spa contexts [30]. The objective of this review is to consider issues related to thermal spa waters within the field of the recreational uses in pools, showing homologies and differences from a public health point of view and perspectives for innovative treatments.

## 2. Spa Trends in the World

The last century saw massive changes and new trends in international health-tourism, where, alongside the traditional health services, thermal-tourism and wellness-fitness became increasingly popular [31,32]. The spa industry has grown by 7.7% annually, from $60 billion in 2007 to $94 billion in 2013, representing the fastest growing subsector in health tourism and leisure sector [33,34]. Several countries reported an increase in spa economy; the spa services in Europe are mostly related to health and healing while spa tourism in the US is more oriented towards the affirmation of a healthy lifestyle [8,31,34]. In this area, Europe maintains a clear leadership; however, the Asia-Pacific region, particularly Thailand, China and Australia, have great potentials and resources for the growth of the wellness spa tourism market, especially due to the price reasonableness of the exclusive services [34,35,36]. Exposure to spa waters and related environments is involving a growing number of people all over the world, posing new question related to safety and public health issues.

**Figure 1:**
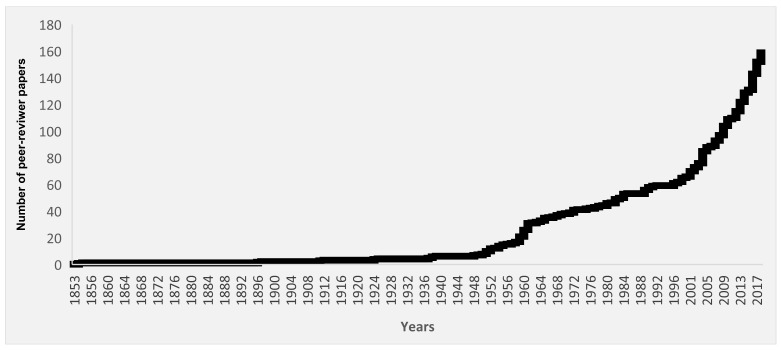
Number of publication entries in Medline (PubMed trend from 1853 to 2018, last access 10/2018). Publication entries were searched with the query: “thermal waters” OR “medicinal waters” OR “spa salus per aquam”.

The increasing interest in thermal waters and spa resources is also reflected in the scientific literature. Indeed, research related to “thermal waters” or “medicinal waters” or “spa salus per aquam” has increased over the last 50 years (Figure 1). The first publication dated 1853 and it already underlined how thermal water properties cannot be not altered in any way by treatments [37]. Later, several authors have investigated the application of thermal water medicine and the nature of spa waters [38,39]. The use of thermal waters for therapeutic purposes has always aroused a continuous interest and debate all over the world, being dependent on the detailed physicochemical pattern of the water joined with the specific indication for a treatment in a defined pathological condition [40,41]. Following the number of publications from different regions, it can be noticed how the interest in this topic is mainly concentrated in Europe (42.3%), Asia (26.3) and Africa (21.7%). Regardless the complex and heterogeneous debate on evidence-based therapeutic applications, spa waters represent a current major approach to wellness worldwide. Their frequent use in pools often is a challenge for public health authorities, both at cultural and technological level. 

## 3. The Question about “Identity” and “Untouchability” of Spa and Medicinal Natural Waters

The growing popularity of swimming and other water activities for sports, fitness, therapy, wellness or relaxation and amusement has increased the diffusion of swimming pools as well as specific-use pools, such as spa pools, hot tubs, whirlpool bath, and natural spa pools [22,25,42]. The term “spa” is an acronym for *salus per aquam*, meaning health through water [5]. The common terms associated with spa pools include hot tubs, whirlpool bath, and natural spa pools; all these types of pools imply different water management and are designed for different wellness, therapeutic or recreational purposes. Most spa pools (>98%) resigned their natural properties, being disinfected by the addition of traditional chemicals with high oxidation potential, such as chlorine [25]. The treatment choice as well as the risk assessment process must consider the nature of the water that supplies the pool plant [25]. In natural spa pools, indeed, the water should remain untreated because the claimed beneficial effects are supposed to derive from their unique chemical-physical properties (Table 1) [22,25,43]. 

**Figure 2:**
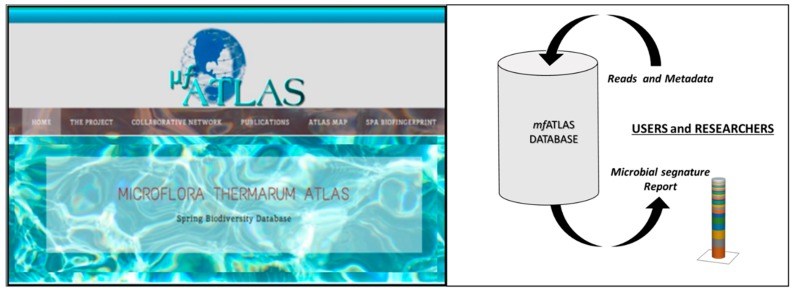
The mfAtlas database: presently, the database is accessible to the research network at www.mfATLAS.it. The database is designed to be further extended to harbour information such as water management, environmental and epidemiological data, international legislations.

Moreover, in order to characterize these spring waters, also the biological component plays a role. Spa waters, indeed, contain a metabolically versatile microflora that is characterized by specialized bacteria belonging to that ecological niche, within a defined range of chemical and physical parameters [43,44]. The biotic and abiotic components of these ecological niches have been deeply studied, representing a mine for the identification of unknown and/or extremophile species within the complex microbial community [44,45,46,47]. Today, it is possible to characterize this microbial community by a massive-sequencing approach, describing a spa water microbiota and defining a “microbial signature” that can be sampled and typed from the original spring source up to the pool facility and final user’s applications [48,49,50]. This approach was already applied in different spa pools and can provide further perspectives for the characterization of spa waters in recreational or wellness uses, adding a candidate new biological parameter to the traditional chemical-physical ones: the microbiota as a novel marker for public health [49,50,51,52]. Interestingly, it is now possible to associate water properties to its microflora component, e.g., presence of H_2_S and communities of sulphate reducing bacteria, temperature and thermophiles, iron (Fe) and iron (III)-reducing bacteria, unravelling biochemical pathways and considering water as an active biological fluid [24,53,54]. The microbiota itself and the analysis of microflora biodiversity by Next Generation Sequencing (NGS), can support the proposal of a modern classification of waters and their properties, opening up new perspectives for the development of appropriate strategies for managing hygiene by respecting chemical, physical and also microbiological natural components [10,50,53,54,55,56]. This approach is very promising but still limited not just by the need of wet-laboratory equipment, protocols or qualified personnel because several external services are available and more and more affordable. Most of all the bottleneck is determined by the requirement of dedicated bioinformatic tools, such as database driven software to analyze the data obtained from massive sequencing and transfer information in an appropriate form to address public health questions. In order to collect and map information from different springs, a dedicated database for spa microbiota was developed and made accessible online to the collaborative research network at www.mfATLAS.it [50]. This tool was designed within a project focused on studying the biological component in spa water springs and pools (Figure 2). It is open to collaborations to analyze and host data from additional sampling points all over the world and the relative metadata, further expanding the atlas-map and database. The availability of this massive sequencing approach and bioinformatic support can improve knowledge on the natural microflora inhabiting thermal spring waters, their geographical distribution, providing also information for the identification of new species and their potential role in the field of wellness, therapeutics in that spa facility, or for other biotechnology applications [11,12,13,14,15,55]. Based on the available version of the mfAtlas database and in according with the observations from other studies and the Earth Microbiome Project, the percentage of unknown species in is still high, covering about 10–70% of the spa water microbiota component [50,56]. Previously, the access to unravelling these complex environmental communities was strongly restricted by the available culture-based methods or classical sequencing of libraries after cloning steps, rather than the massive sequencing approach that today is rapidly and successfully diffusing in different fields [56,57,58,59,60]. NGS revealed as a promising strategy not only in characterizing the natural microflora of spa waters, but also the presence of microbiological markers, pathogens or the effectiveness of disinfection and other water treatments [56,57,58,59]. This novel approach to solve hygiene questions is based on the genetic analysis of water biodiversity, starting from the DNA of its microflora (mfDNA) [52]. It is opening promising perspectives for understanding the beneficial potentials of spa waters, their fingerprint and their “untouchability” based on the respect of chemical, physical, and also biological components.

### Treatments for Spa and Medicinal Natural Waters: Limits and Perspectives

Several alternative water treatments were considered to assure the original properties and composition of the water, reducing the adverse effects on bathers and environments, to the final aim of offering sustainable solutions for spa waters in pools [61,62,63]. This challenging objective shares principles and problems also with the management of other kinds of waters, so that a success in this field can impact in other areas of water hygiene and conversely.

Otherwise, traditional disinfection approaches, e.g., chlorine, not only affect the harmful microorganisms present in the pool but also destroy the beneficial chemical-physical composition and the natural microflora, adulterating the therapeutic proprieties of these waters. The organic and inorganic compounds present in these waters can react with chemical substances used for disinfection, such as chloride/bromide-based chemicals, generating potentially toxic secondary products [24,63,64,65,66]. Nonetheless, the equilibrium between maintaining the natural proprieties of the thermal waters in pools and minimizing the microbial risk for people is not easy and can be achieved by considering several candidate approaches [22,54,66]. Firstly, a possible solution can be based on a personalization of the hygiene approach. The waters for natural spas, indeed, should be carefully tested for satisfactory microbiological quality before designing and constructing the water-plant, adopting a kind of individualized strategy based on the specific composition and destination use of that water in that plant, following a dedicated water safety plan [10,22,25,66,67,68,69]. In vitro models and protocols can prove useful in comparing materials or treatment methods [70]. The main task of water management is to achieve a satisfactory control on exposure of the bathers to infectious agents or other health risks, realizing an effective prevention of disease and accidents [22]. The high salinity or high temperature of spa waters represents an additional challenge for the pipeline plant and its maintenance, e.g., due to corrosion and concretion. Managing natural spa pools should follow the guidelines for traditional pools, but additional concerns have to be highlighted, so that alternative strategies may also be considered if proven to be effective and acceptable [25,47,68,69,70].

Even if spa waters should not be treated in order to try to maintain their original properties, however, sometimes they are used as a common source of water for just filling a pool for recreational purposes. Regarding the use of traditional pool disinfection of spa waters e.g., by chlorination, several inconveniences can occur in addition to the generation of known but also of unexpected Disinfection by-products (DBP) [65,66,69]. DBP are derived after the interaction with organic materials following an already well-known process, but the scenario is complicated by the presence of other chemicals naturally presents in the spa water or introduced by the bathers [22,65,71]. Oxidation in presence of an already high salinity can favor precipitates and concretions or induce unexpected toxic DBP with undesired -and often unpredictable-effects on bathers [71]. Moreover, the high temperature and the intense aeration due to frequent bubbling in this kind of pools, considerably can increase the evaporation of the active chemicals. Therefore, the disinfectant active doses are often poorly quantified, and “classic” chemicals generally may show a very irregular efficiency in spa pools. The type, form and use of each disinfectant need to be chosen with respect to the specific requirements of the pool [68,71,72,73,74]. Pool size itself may represent a critical parameter [22,25]. The choice of the disinfection strategy must be made after consideration of the efficacy of the specific product under the specific circumstances of use and the feasibility of a monitoring of the disinfectant levels in that pool [22,25,75,76,77]. Table 2 lists the several types of disinfecting agents and their advantages and limits of use in swimming pools as well as their applications in spa pools. Chlorine is inexpensive and relatively convenient to produce, store, transport, and apply. It provides rapid and long-lasting bactericidal effects but is limited mainly because of the formation of potentially toxic DBPs, such as trihalomethanes, halomethanes (THMs), haloacetic acids, halonitromethanes, haloacetonitriles, chloramines, and chlorophenols [22,25,65,72,73,74,75,76,77,78,79,80]. For example, the levels of potentially toxic DBPs tend to be higher in hot tubs, due to recirculation and smaller volumes but also because acceptable thresholds tend to be more elevated than in swimming pools [73,74,75]. However, when treating natural spring waters with chemical disinfection, whatever the final use, their natural characteristics are modified [22,25]. In order to avoid adulteration of the natural properties of spa pools, a commonly adopted alternative is based on dilution of pollutants by the frequent replacement of pool water. This may be feasible for small pools and when a large reservoir is available, but it can become unsustainable on the long term, due to the risk of depleting the aquifer. 

Ozonation or ultraviolet (UV) irradiation represent additional solutions that are known and already well engineered and tested. Even if effective they can be demanding to maintain during time and both methods have no residual disinfection activity in the pool water [80,81,82,83,84,85,86,87,88,89]. Recently, advanced oxidation processes (AOPs) have shown a demonstrated efficacy in the treatment of organic pollutants in aquatic environments, but AOP technologies involve the generation of nonspecific hydroxyl radicals and the production of activated compounds involved in THM formation in the post-chlorination step, thus increasing the potential for DBPs formation [61,76,77,81]. UV irradiation is effective for controlling resistant microorganisms, such as *Cryptosporidium parvum* and *Giardia lamblia* [83,84,85]. This physical treatment seems cost-competitive in terms of improving the quality of swimming pool water; however, it has several limitations, including the life spam of the lamp and the potential formation of nitrogenous-based DBPs [61,82,83,84,85,86,87,88,89,90,91]. Bromine-based disinfectants may provide rapid and long-lasting disinfection effects, but they are more difficult to manage [61,92]. Several studies raised the problem of DBPs and reported eye or skin irritation due to bromine-based disinfectants [92]. The use of bromine-based disinfectants is generally not very feasible for outdoor pools and spas also because the bromine residue can get rapidly depleted in sunlight [25,92]. Copper/silver ionization was also proposed based on experimental observations on the effectiveness of silver nanoparticles (NPs) on harmful microorganisms, but several limits were reported including toxicity [61,92]. Hydrogen peroxide is a broad-spectrum disinfectant usually supplied as a solution to be dosed or added to spa pools; it is generally prepared by stabilizing ion-based chemistry [93]. The limitation in using hydrogen peroxide is the requirement of high concentrations depending on the condition of the facility; therefore, hydrogen peroxide disinfection was suggested only for small pools [25].

The World Water Development Report 2018 has outlined innovative natural treatments [94]. In this document nature-based solutions (NBS) are defined as a potential contribution to solving or overcoming the major water management problems or technical challenges [22,25,95,96,97,98]. A revolution in water treatment technologies is occurring and novel treatments based on physical methods are now considered and studied [96,97]. Membrane filtration has largely replaced granular filtration, and UV irradiation is enabling reduction in the use of or even elimination of classic disinfection chemicals, such as chlorine and its derivatives [97,99,100]. Ultrafiltration membranes are widely used in water treatment because of their favourable characteristics, such as easy modularization and improvement in water quality. A main limitation is membrane fouling that induces a reduction of membrane flux, an increase in energy consumption and in the consequent costs for water treatment [98].

Rapid advances in nanotechnologies have encouraged the development of industrial applications of manufactured nanoparticles (NPs) in a wide range of commercial products, such as drugs, paints, electronics, foods, or cosmetics [101]. However, laws or guidelines regarding the applications of NPs still are lacking and additional research on safety performance standards is required [101,102]. Table 3 summarizes the main innovative strategies and the possible applications of NPs in spa pools. For example, nanoscale chitosan and its derivatives were proposed also in water treatment, because of their antimicrobial effects on bacteria, viruses, fungi, and bacteriophages, through damaging cell membrane or chelating trace metals [101,102,103,104,105,106]. Chitosan is currently used in personal care products and biomedical products, as microbicide in agriculture and food wraps, and as a flocculant in water and wastewater treatments [102,103,104,105,106]. It is a promising compound for low-cost and low-tech disinfection applications and it was suggested for applications in developing countries, but it has several limitations including its deterioration under different conditions [107]. A promising scenario is coming from the use of light of different wavelength through photocatalysis processes [108]. In principle, this approach can allow water treatment just by light and air, through the production of free radicals. Metal oxide semiconductor NPs with a wide band gap are the basic materials used in heterogeneous photocatalysis method. They can accelerate the degradation of pollutants under solar illumination [108]. Titanium dioxide (TiO_2_) and zinc oxide (ZnO) NPs are among the most extensively used metal NPs [107,108]. TiO_2_ is already present in different products including foods additives and cosmetics, paints and also coating for pools [70,109]. Silver and copper metals have been also considered for their antibacterial properties within nanotechnological applications. Other kind of nanoparticles, Silver NPs (AgNPs), have been developed since late 1800s and have been registered with the Environmental Protection Agency for use as swimming pool algaecides since 1954 and as drinking water filters since 1970s [108,109,110,111].

AgNPs exhibit a strong and broad-spectrum antimicrobial activity and showed no harmful effects on humans [112]. They are currently being used in the development of point-of-use water disinfection systems and antibiofouling surfaces [113]. An innovative scenario is offered by the availability of Carbon nanotubes (CNTs), that have been reported to induce DNA damages and cytotoxic effects in prokaryotic cells, consequently disrupting the microbial diversity and community structure [114]. Several possible toxicological mechanisms of CNTs on microorganisms have been proposed, among which the disruption of the cell membrane integrity, that is considered a key mechanism in this antimicrobial process [114,115,116]. In the 21st century, advances made in the synthesis of carbon-based nanomaterials have resulted in the development of graphene–carbon nanotubes [117]. Notably, the three-dimensional graphene and graphene oxide-based nanostructures exhibit a large surface area and sorption sites that provide a high adsorption capacity to efficiently extract pollutants and inactivate viruses or bacteria in water [118,119].

In conclusion, advancements in the field are in continuous progress and several alternatives have been proposed for water treatments, opening promising perspectives also for thermal spa pools. Due to the heterogeneity of thermal spa waters and their peculiar requirements for the therapeutic use in spa facilities, presently it seems still difficult to find a single strategy for all different situations. Therefore, more than a unique ideal solution, the optimal strategy should be searched in the combination of different methods, following an individualized approach based on water properties, plant characteristics, destination use of the spa pool.

## 4. Guidelines and Regulations on Thermal Spa Water Pools

The global scenario of the international regulations in the spa field is very heterogeneous and reflects the socio-economics and culture from the different countries (Table 4). In the USA, local and state regulations consider the routine inspection of aquatic environments for preventing risks and accidents [120]. Deaths due to pool entrapment have led to the enactment of the Virginia Graeme Baker Pool and Spa Safety Act [121,122]. The act outlines provisions to minimize the risk of entrapment, including mandatory requirements with respect to vacuum covers, pool barriers, and main drain requirements. For hygienic aspects, U.S. states have separate law or guidelines (e.g., Alabama and Kansas), although the point of reference remains the guidelines issued by the World Health Organization (WHO) [22,122]. In fact, since 1994, WHO had been promoting the development of guidelines for the use of recreational waters; these guidelines have now evolved to safety guidelines for recreational aquatic environments [22]. In Canada, British Columbia health authorities approve and inspect pools, hot tubs and other facilities to ensure safety in construction and operation, according to the Pool Regulation under the Public Health Act [123]. Hygienic-sanitary safety of thermal plants is addressed in the 2007 legislation, which has established the quality criteria for waters, including spas, and obligations for pool managers, following the Health Canada’s Guidelines for Canadian Recreational Water Quality. These guidelines address potential health hazards, such as infections transmitted by disease-causing microorganisms, as well as aesthetics and nuisance conditions [124]. The Pool Standards have set specific technical thresholds pertaining to water quality and facility operations requirements under the Public Swimming Pools Regulation as well as other requirements, including: operator and user education, recirculation systems, water chemistry and microbiology indicators, water quality monitoring, anti-entrapment policies and other plans related to pool safety [125]. The standards also include a protocol for the management of contaminated public swimming pool water and the calculations for maximum bather load and flow rates through anti-entrapment suction outlets. These standards were developed in consultation with the pool industry, pool operators, and public health officials and recently revised in 2018 [125]. Australian swimming pool regulations was established in 1990 and revised in 1992, focusing also on spa pools, swimming pools and similar environments but does not include baths [126,127]. In the recent years, some regulations and technical standards have been introduced with regard to spas [128,129]. Europe is a rich continent in terms of natural hot springs, harbouring over 5000 springs and facilities, very popular since ancient times [8,56]. However, the European legislative situation in this matter seems very fragmented and inconsistent. A comprehensive community directive is still missing. The European reference 76/160/EEC concerning bathing water, as amended by 2006/7/EC, is not applicable to the spa and swimming pool waters or to the confined waters subjected to treatment or used for therapeutic purposes [130,131]. Austria had already issued regulations concerning swimming pools with the goal of preventing the spread of waterborne diseases [132]. Subsequent changes extended the scope of the legislation, but only in 2012 a new law has been passed regarding the technical and operational requirements for the water quality of traditional pools and bathing water, whirlpools, small natural pools, and ancillary facilities used for recreational or therapeutic activities [133,134,135,136,137]. The government of the Brussels-Capital region and the Walloon region in Belgium have issued specific ordinances on sectoral conditions related to swimming pools, saunas, and general artificial reservoirs designed for therapeutic, recreational, or sporting activities, but not domestic facilities [138,139,140]. In France, the current legislative framework related to swimming pools is based on the Public Health Code about the care and rehabilitation in thermal pools with an independent section concerning traditional pools [141,142]. Furthermore, in 2010, the Agency for Environmental Health and Safety (ANSES) published a document on health risks in swimming pools, indicating the thermal pools as “atypical” pools [143]. In 2013, ANSES published the part II of this document, focusing on hot tubs (“bains à remous”) [144]. In England, Health and Safety Executive is the authority overlooking the pools of local authorities and schools. This institute together with the Health Protection Agency published the guidelines concerning the control of the risk of infections in thermal pools [145]. These protocols have been designed to improve the understanding about microbiological risks associated with the use of thermal baths and to provide advice on risk management [25,145]. Similarly, in Ireland, a country with a large number of geothermal pools, every swimming pool is a public spa that is managed in compliance with the Safety, Health and Welfare at Work Act of 2005 as well as a specific set of guidelines published to provide administrators with criteria and detailed information for management [146,147]. In Germany, the technical provisions for the management of swimming pools are summarized in the DIN 19643, which guarantees hygiene safety in swimming pools, saunas, whirlpools, and spas [148,149]. Periodically, the Federal Environment Agency publishes recommendations for health managers and authorities [150]. Recently, new developments in swimming pool hygiene required a revision of the standard series DIN 19643, with the introduction of new treatment processes based on ultrafiltration [151]. Czech Republic has a legislation concerning the spa sector, but it is not extended to the recreational applications of these waters or to the sanitary-hygiene related issues [152]. More specific standards concerning the management of water for therapeutic uses and thermal spas, are also available [153,154]. Iindependent standards are available in Portugal for specific regulations in spa facilities, focusing on licenses, organization, management, and control and attention is dedicated to the quality of public pools. However, Portugal uses a directive issued by the Council National Quality that does not apply to thermal pools for therapeutic use, indicating to other specific regulations [155,156]. In Slovakia, a country with a great spa tradition, water quality used for swimming is managed by the Public Health Authority and 36 regional health authorities, which overlook the pools supplied with thermal water [157]. In Spain, pool regulations are available since 1987 and the health authorities refer to the WHO guidelines [158,159,160]. The Ministry of Health, Social Services and Equality of Spain has developed the Real Decreto project to establish the water quality criteria for public pools, spas, private pools, and water parks but excludes natural pools and thermal waters used for medical therapeutic purposes [161]. An innovative appearance of this law is the consideration of water safety plans. Finland has included recommendations for public baths, spas, and swimming pools in the application law for the European directive n.2006/7/CE [162,163]. Other countries, namely Cyprus, Bulgaria, and Norway, have considered a different approach, not including this 2006 Directive along the specific rules for pools [164,165,166]. In Italy, the recreational use of spas has extensively increased in the last few years, but no specific guidelines have been established, yet. In fact, more recently, the Italian legislation started referring to the law of “the reorganization of the thermal system” established in 2000 for thermal waters [167]. This law reports the definition of thermal waters and the provisions concerning bottling and permitted uses but does not deal with the hygiene aspects related to the recreational use. The current legislation concerning swimming pools is the January 16th Agreement 2003 between the Minister of Health, the Regions, and the Autonomous Provinces of Trento and Bolzano [168]. This document is under revision and a public consultation was performed in 2016 [169]. The Agreement specifies that the swimming facilities can be supplied with different types of water, including thermal waters, but postponing the discipline of the latter to specific regional measures; moreover, additional guidelines for the spa hygiene are available for preventing Legionnaire’s. This document has a chapter fully dedicated to swimming pool measures that underlines the need for adequate design of pool spa facilities because no specific treatment of these waters is allowed [170]. In summary, the complexity of the argument in the different countries does not seem to have fostered the development of unequivocal rules and shared strategies. Future joined projects and consensus documents would be welcome and useful on a local and international scale.

## 5. Conclusions

Hot spring waters represent a unique natural fluid that humans have used since ancient times for health and recreational purposes. Spa facilities are present all over the world denoting a relevant resource for business that involves a large and growing number of users. The safeguard of the natural composition of spa waters clashes with the need of appropriate treatments in pools. Innovative strategies have been proposed, but further studies and validations are required. In addition to traditional chemical-physical parameters, the possibility to characterize the biological component is opening new perspectives for the classification and fingerprinting of spa waters through mfDNA (microflora DNA) analysis and the definition of spa microbiota patterns. Recent advancements in massive sequencing and bioinformatics are supporting this process, providing new tools for hygiene and knowledge on properties of spa water. The heterogeneity of spa waters and their uses may suggest an individualized approach to design and carry on a sustainable management through dedicated technical solutions and water safety plans. Public health regulations for the use of spa waters in pools are mainly lacking and a consensus at international level would be needed and welcome for providing agreements and shared guidelines.

## Figures and Tables

**Table 1:** Classification of natural mineral waters based on fixed residue at 180 °C and chemical composition, according the 2009/54/EC Directive [43].

Classification of Mineral Waters	
Classification according to fixed residue at 180°	Classification according to chemical composition
Very low mineral content waters (Fixed residue <50 mg/L)	Bicarbonate waters (>600 mg/L)
Low mineral content waters (Fixed residue 50–500 mg/L)	Calcic waters (>150 mg/L)
Medium mineral content waters (Fixed residue 500–1500 mg/L)	Chloride waters (>200 mg/L)
Rich mineral content water (Fixed residue <1500 mg/L)	Ferrous waters (>1 mg/L)
	Fluorurate waters (>1 mg/L)
	Magnesiac waters (>50 mg/L)
	Sulphated waters (>200 mg/L)
	Sodium-Rich waters (>200 mg/L)

**Table 2:** Several types of antimicrobial agents and their candidate applications in SPA pools: main advantages and limits for swimming pool uses.

Disinfection Solution	Advantages	Limits	SPA Pool Applications	References
Chlorine-based disinfectant	Inexpensive and relatively convenient to produce, store, transport and use. Provides rapid and long-lasting disinfection effects. Residual disinfectant activity in pool.	The formation of potentially toxic DBPs, such as THMs, HAAs, HANs, THAs and CAMs. Presence of chlorine-resistant microorganisms such as *Cryptosporidium parvum* and *Giardia lamblia*.	In hot tubs, acceptable free chlorine levels tend to be higher than in swimming pools. Moreover, due the chemical characteristics of thermal water, the reaction between chemical compound and disinfection agents can lead the increase the potentially toxic DBPs.	[22,25,65,72,73,74,75,76,77,78,79,80]
Ozone	Highly effective, no smell. Can reduced the formation of potentially toxic disinfection by-products (DBPs).	Toxic and explosive; heavier than air. Risks and adverse health effects for the operator. Lack of residual disinfection proprieties; (usually joined with chlorine). Production of activated compounds suitable for THMs formation in the post-chlorination step.	AOPs have recently shown successes in the treatment of organic pollutants in aquatic environments, involving the generation of non-specific OH radicals. A de-ozonization step is needed.	[61,81]
Ultraviolet (UV) irradiation	Physical treatment without adding chemicals to the water. Effective for the control of resistant microorganisms including protozoa such as *Cryptosporidium parvum* and *Giardia lamblia*.	The formation of nitrogenous-based DBPs (HANs) Lack of residual disinfection proprieties.	UV radiation can be proposed to reduce the risk of infection by dermatophytes eventually present in swimming pools that use thermal water. Cost-competitive with chlorine to improve the quality of swimming pool water.	[61,81,82,83,84,85,86,87,88,89,90]
Bromine-based disinfectant	Inexpensive and relatively convenient to apply. Provides rapid and long-lasting disinfection effects.	It is difficult to dissolve and must be inserted into the pool through an automatic feeder. DBP There are reports that is associated with eye and skin irritation.	The use of bromine-based disinfectants is generally not practical for outdoor pools and spas because the bromine residual is depleted rapidly in sunlight.	[25,61,92]
Stabilised silver/copper	Copper/silver ionization was proposed for treatment of swimming pool water: protocols and devices are available. No pH adjustment is required.	Low effectiveness Limited information on toxicity of ion forms and interaction with other chemicals.	Silver is a broad-spectrum disinfectant usually supplied as a solution to be dosed or added to the spa-pool system. Higher concentrations may be required depending on the condition of the facility.	[25,61,92]
Hydrogen peroxide	Effective Low pollution on water.	With hydrogen peroxide the by-products are not problematic but it can generate toxic radical compounds.	Hydrogen peroxide can be used with silver and copper ions (low levels of the silver and copper): proper consideration to replacement of water for preventing excessive build-up of the ions.	[25,93]

Note: Disinfection byproducts (DBPs); Hypochlorous acid (HOCl); Trihalomethanes (THMs); Haloacetonitriles (HANs); Hydroxyl radical (OH); Advanced oxidation processes (AOPs).

**Table 3:** Current and potential applications of antimicrobial nanomaterials.

Nanomaterials	CAS NUMBER	Nature of Disinfection Type		Antimicrobial Mechanism	Current Applications	Potential Future Applications in SPA Pools	References
		*Physical*	*Chemical*				
**Silver nanoparticles (AgNPs)**	7440-22-4		☑	AgNPs can disrupt the outer membrane of target cells.	Portable water filters, clothing, medical devices, coatings for washing machines, refrigerators, and food containers	An alternative to traditional chemical disinfectants that are prone to generate harmful disinfection by-products	[101,108,109,110,111]
**Chitosan**	9012-76-4		☑	Membrane damage, chelation of trace metals. Nano-scale chitosan and derivatives exhibit antimicrobial effects towards bacteria, viruses, fungi.	Personal care products, microbicide in agriculture and biomedical products, food wraps, biomedical, flocculants in water and wastewater treatment	They are promising for low-cost and low-tech disinfection applications. In water filtration, chitosan combined with sand filtration removes up to 99% of turbidity.	[101,102,103,104,105,106]
**Graphene oxide**	1034343-98-0	☑	☑	DNA damages and cytotoxic effects towards prokaryotic cells and detrimentally change the microbial diversity and community structures	Graphene oxide (GO) and silver-graphene oxide (Ag-GO) are used in various fields, such as biotechnology and environmental engineering, due to their unique material properties, including hydrophilicity, high surface area, mechanical strength, and antibacterial activity	In aquatic ecosystems, the stability of nanomaterials is affected by the water chemistry parameters of the receiving aquatic environments such as ionic strength, natural organic matters and pH	[114,115,116,117,118,119]
**H_2_S**	7783-06-4		☑	H_2_S killed microorganisms through inducing oxidative stress by inhibiting antioxidant enzymes	None	Restore the normal bacteriostatic nature of the thermal water	[24]
**Nano TiO_2_**	13463-67-7	☑	☑	Production of Reactive Oxygen Species (ROS), cell membrane and cell wall damage	Air purifiers, water treatment systems for organic contaminant degradation.	The applicability is in evaluation. The presence of some inorganic ions can be problem, because reduce the performance of TiO_2_ in water treatment.	[108,109]
**Ultrafiltration**	-	☑		Ultrafiltration allowed the removal of suspended matter, as well as a part of the organic matter	Water treatment, swimming pool	Ultrafiltration can be selected as an alternative treatment process because of its ability to remove bacteria and viruses.	[97,98,99,100]
**ZnO**	1314-13-2	☑	☑	Intracellular accumulation of nanoparticles, cell membrane damage, H_2_O_2_ production, release of Zn^2+^ ions	Antibacterial creams, lotions and ointment, deodorant, self-cleaning glass and ceramics	Surface coating	[108,109]

**Table 4:** International guidelines, regulation and recommendation regarding recreational water environments.

Country	Law	References
***Australia***	New South Wales Consolidated Acts. Swimming Pools Act 1990 n. 31.	[126,127,128,129]
	New South Wales Consolidated Acts. Swimming Pools Act 1992 n. 49.	
	Standard. Spa Pools Part 1: Public spas. 2007	
	Pool Water Quality and Operational Guidelines.	
***Austria***	Bundesgesetzblatt für die Republik Österreich 1978; 167:3053–63.	[132,133,134,135,136]
	Mitteilungen der Österreichischen Sanitätsverwaltung, 1992;93(11):358.	
	*Bundesgesetzblatt für die Republik Österreich*. 1996; 212:4617-24.	
	*Mitteilungen der Österreichischen Sanitätsverwaltung*. 1997;98(5):228–32.	
	Gesamte Rechtsvorschrift für Bäderhygienegesetz, Fassung vom 28.10.2012.	
***Belgium***	Belgio. Arrêté du Gouvernement wallon portant conditions sectorielles relatives aux bassins de natation.	[138,139,140]
	Belgio. Arrêté du Gouvernement de la Région de Bruxelles-Capitale fixant des conditions d'exploitation pour les bassins de natation.	
	Belgio. Arrete´ du Gouvernement de la Region de Bruxelles-Capitale fixant la liste des installations de classe IB, II et III en execution de l’article 4 de l’ordonnance du 5 juin 1997 relative aux permis d’environnement.	
***Bulgary***	*D’rzaven vestnik* 1994; 65:1–14.	[165]
***Canada***	Règlement de sécurité, Fédération de natation du Québec (natation en bassin)	[123,124,125]
	Guidelines for Canadian Recreational Water Quality	
	Alberta Health Pool Standards	
***Ciprium***	*Ciprium Government Law N. 55(I)/92*	[164]
***Czech Republic***	Decree of Ministry of Health No.423/2001—On Spas and Sources	[152,153,154]
	Decree of Ministry of Health No.252/2004—Requirements on Cold and Hot Water in Health Care and Accommodation Facilities	
	Decree of Ministry of Health No.135/2004—Requirements on Swimming Pools, Saunas and Outdoor Playgrouds.	
***Finland***	Finlands Författningssamling 2008/70.	[162,163]
	Finlands Författningssamling *2014/47*	
***France***	Code de la santé publique, 2010. Section V: Surveillance des établissements thermaux.	[141,142,143,144]
	Code de la santé publique, 2010. Section I: Normes d'hygiène et de sécurité applicables aux piscines et baignades aménagées	
	Afsset Evaluation des risques sanitaires liés aux piscines Partie I: piscines réglementées. Saisine Afsset «2006/11». Rapport final. 2010	
	Anses. Évaluation des risques sanitaires liés aux piscines Partie II: bains à remous. 10.13140/RG.2.1.2182.7043.	
***England***	Management of Spa Pools: Controlling the Risk of Infection. Health Protection Agency. March 2006.	[25,145]
	Health and Safety Executive (HSE). The control of Legionella and other infectious agents in spa-pool systems.	
***Germany***	DIN 19643. Aufbereitung von Schwimm- und Badebeckenwasser–Teil 1: Allgemeine Anforderungen.Beuth,Berlin	[148,149,150,151]
	Hygienische Anforderungen an Kleinbadeteiche. Empfehlung des Umweltbundesamtes. Bundesgesundhbl	
	Bundesgesundheitsbl-Gesundheitsforsch-Gesundheitsschutz	
	DIN 19643. Aufbereitung von Schwimm- und Badebeckenwasser—Teil 4: Verfahrenskombinationen mit Ultrafiltration	
***Italy***	Law of 24 October 2000, n. 323. Reorganization of the thermal sector. Official Gazette November 8, 2000, n. 261.	[167,168,169,170]
	Agreement between the Minister of Health, the Regions and the Autonomous Provinces of Trento and Bolzano G.U. March 3, 2003: 45, n. 51.	
	Guidelines with indications on legionellosis for managers of tourist accommodation and thermal facilities G.U. n 28 Febrary 5, 2005	
***Ireland***	Safety, Health and Welfare at Work Act”, 2005. Health and Safety Authority	[146,147]
	Swimming Pool Safety Guidelines. Irish Water Safety, ILAM and Swim Ireland. 2010.	
***Norway***	*Norsk Lovtidend*, 1 sezione. 1996;11:767–73.	[166]
***Portugal***	Ministério da saúde Decreto-lei n. 142. 11 giugno 2004	[155,156]
	Directiva Conselho Nacional da Qualidade "A qualidade nas piscinas de uso público". n.º 23, 1993.	
***Slovakia***	*Zbierka zàkonov Slovenskej Republiky* 1994;77:1350-1370.	[157]
***Spain***	Boletìn Oficial del Ministerio de Sanidad y Consumo 1987;19:1147-52.	[158,159,160,161]
	Boletìn Oficial del Ministerio de Sanidad y Consumo 1998, 80. por el que se regulan las condiciones higiénico–sanitarias de piscinas de uso colectivo.	
	Boletin Oficial orden 1319/2006	
	Real Decreto 742/2013	
***USA***	CDC's Model Aquatic Health Code	[120,122]
	Virginia Graeme baker Pool and Spa Safety Act	
	Dedicated law and guidelines for U.S. STATES	
